# Infrared thermography and dual-layer spectral CT for the detection of femoral vascular compromise: an experimental large animal study

**DOI:** 10.1007/s00068-026-03237-x

**Published:** 2026-06-30

**Authors:** Christian Thomas Hübner, John Ricklin, Alina Leoni Müller, Felix Karl-Ludwig Klingebiel, Christian T. Stoeck, Miriam Weisskopf, Alice Carneiro Gomes, Paolo Cinelli, Hans-Christoph Pape, Roman Pfeifer, Yannik Kalbas

**Affiliations:** 1 Department of Traumatology, University Hospital Zürich, Raemistrasse 100, Zürich, 8091 Switzerland; 2 Harald-Tscherne Laboratory for Orthopaedic and Trauma Research, University Hospital Zurich, University of Zurich, Raemistrasse 100, Zurich, 8091 Switzerland; 3 Center for Preclinical Development, University of Zurich and University Hospital Zurich, Raemistrasse 100, Zurich, 8091 Switzerland

**Keywords:** Infrared thermography, Spectral CT, Vascular compromise, Hemorrhagic shock, Polytrauma

## Abstract

**Background:**

Rapid identification of vascular compromise remains a clinical challenge in the acute trauma setting. Conventional diagnostic tools such as CT angiography (CTA) and Doppler-ultrasound might provide decent anatomical and functional assessment. However, they are limited by ionizing radiation, contrast exposure or time requirements. Although not validated, infrared thermography (IRT) might offer a non-invasive alternative capable of detecting surface temperature asymmetries that may reflect underlying perfusion deficits. This study aimed to evaluate the feasibility of thermographic imaging in detecting vascular lesions.

**Methods:**

As part of a porcine polytrauma model (*n* = 28), vascular lesion of the right femoral artery was defined as a ≥ 50% diameter reduction on CTA. Temperature differences between both extremities were measured using infrared thermography. SDCT based iodine concentrations distal to the stenosis were quantified to assess perfusion changes. Correlation and regression analyses were performed between stenosis degree, temperature difference, and tissue iodine uptake.

**Results:**

Six animals fulfilled the criteria for relevant vascular compromise. This resulted in a mean vessel diameter reduction behind the lesion of 68% ±18%. The vascular lesion group demonstrated a mean inter-limb temperature difference of 6.93 °C ± 1.78 °C compared to 0.71 °C ± 1.27 °C in the non-lesion group (*p* < 0.001). A strong positive correlation was found between diameter-based stenosis and temperature difference (*r* = 0.785, *p* < 0.001), as well as between diameter reduction and iodine uptake (*r* = 0.801, *p* < 0.001). ROC analysis of temperature difference for detecting vascular lesion yielded an AUC of 0.992 (95% CI 0.970–1.000, *p* < 0.001) with 100% sensitivity and 95.2% specificity at a 3 °C threshold.

**Conclusion:**

Thermographic temperature differences showed excellent diagnostic performance in detecting an acute vascular lesion in a large animal model. The strong correlation between SDCT iodine uptake and both anatomical stenosis and surface temperature suggests that iodine concentration is a valid quantitative surrogate for perfusion. In conclusion, infrared thermography might represent a viable alternative for detection of acute vascular occlusion.

## Introduction

Vascular occlusion or lesions are clinically relevant findings in trauma patients, especially after polytrauma [1]. Rapid and reliable detection of vascular compromise in the acute trauma setting remains a clinical challenge [2]. Traditionally, clinical signs such as the “six Ps” (pulslessness, pallor, pain, paresthesia, paralysis, poikilothermia) can be assessed. However, these examinations depend on the experience of the examiner and have a limited interobserver reliability [3, 4]. Furthermore, some of these findings are only reliable in later phases of vascular damage (e.g. pulselessness) [5]. In addition to this physical examination, ankle-brachial index (ABI) can be used to support diagnostic assessment, even though this is not feasible in the acute setting in polytrauma patients [6–9].

In recent years, computed tomography angiography (CTA) has emerged as a reference standard imaging modality for diagnosing vascular injuries in trauma patients, offering detailed anatomical visualization of arterial stenosis, occlusion, and other vascular pathologies [10, 11]. As rapid whole-body CT imaging is frequently required in trauma patients, CTA offers substantial potential for early diagnosis [12, 13]. However, CT angiography is associated with exposure to ionizing radiation, potential nephrotoxicity from iodinated contrast agents and may be critical in unstable patients [14, 15]. Furthermore, standard whole-body CT protocols extend from the head to the proximal femora and often lack coverage of the peripheral extremities.

A practical alternative or adjunct may be vascular ultrasound. It provides a rapid, bedside assessment of arterial flow and is a portable and radiation-free technique [16]. However, comprehensive duplex mapping can be time-consuming and operator-dependent, particularly in distal vessel assessment [17]. Due to its long assessment duration, it is often not feasible in the acute care setting.

Measurements with infrared thermographic cameras could represent a promising non-invasive, non-contact imaging modality. By detecting emitted infrared radiation from the skin surface, it converts this radiation into distinct temperature values. This offers the possibility to generate a colorized thermal map representing the spatial distribution of surface temperatures. Some studies have investigated thermal imaging in patients with Peripheral Artery Disease (PAD) or after vascular surgery [18–20]. In these settings, IRT may be regarded as a tool to objectively visualize and quantify poikilothermia [21]. In the literature, temperature differences of 1–3 °C when comparing both sides are classified as clinically relevant. Limbs with arterial insufficiency demonstrate measurably lower surface temperatures compared to adequately perfused extremities [20]. These findings support the concept that inter-extremity temperature differences can serve as a practical screening tool for vascular compromise in trauma patients.

As part of a large animal model, investigating alterations after severe polytrauma, we assessed whether thermographic imaging is feasible to detect vascular occlusion after tissue trauma and/or hemorrhagic shock. Furthermore, it should be tested, if flow measurement using Dual-layer spectral CT (SDCT) is a valid parameter and correlates with vascular occlusion.

## Materials and methods

The current study was conducted as part of a standardized porcine model investigating posttraumatic alterations in polytrauma and shock [22]. All procedures were approved by the local veterinary office (Cantonal Veterinary Office, Zurich, Switzerland, No. ZH069-2024) and conducted in compliance with the Swiss Animal Protection Law, the European Directive 2010/63/EU, and the Guide for the Care and Use of Laboratory Animals (National Research Council, 2011). A detailed overview of the project procedures has been previously described [22]*.*

In summary, thirty-two healthy Swiss Landrace pigs (Sus scrofa domesticus, mixed sex) from disease free barrier breeding were used. Animals were acclimatized for 7 days prior to the experiment under controlled conditions (21 ± 3 °C, 50% humidity). Anesthesia and monitoring were performed according to previously validated large animal trauma protocols. In brief, sedation was induced with an intramuscular injection of ketamine (15 mg/kg), Azaperon (2 mg/kg), and Atropine (0.05 mg/kg), followed by intravenous administration of propofol (3 mg/kg) for induction and endotracheal intubation. Anesthesia was maintained with a continuous rate infusion of propofol, ketamine, midazolam, and sufentanil. Animals were mechanically ventilated in a volume-controlled mode (6–8 mL/kg tidal volume, PEEP ≤ 8 mmHg) with normocapnic targets (PaCO₂ 35–55 mmHg). Ringerfundin^®^ was infused continuously (21 mL/h). Catheterization was performed in a supine position. An arterial catheter was placed ultrasound guided in the right femoral artery. Furthermore, catheterization of the following vessels was performed: right external jugular vein and left femoral vein. Hemodynamic parameters, oxygenation, and ventilation values were continuously monitored throughout the experiment.

### Experimental groups and hemorrhagic shock induction

Animals were randomized into four groups as described elsewhere: isolated hemorrhagic shock (HS), isolated tissue trauma (TTFx), combined polytrauma (PT), and sham (SH). For the present analysis, data from all groups were pooled, and animals developing relevant vascular compromise were compared to those without compromise. Trauma induction followed the published protocol. In groups PT and HS, a hemorrhagic shock by controlled blood withdrawal to maintain a mean arterial pressure (MAP) of 30 ± 5 mmHg for 90 min was induced. Resuscitation was performed after 90 min following ATLS^®^ and AWMF-S3 trauma guidelines, including reinfusion of autologous blood and warmed crystalloids (Ringerfundin^®^, three times calculated blood loss).

### Exclusion of animals

**Fig. 1:**
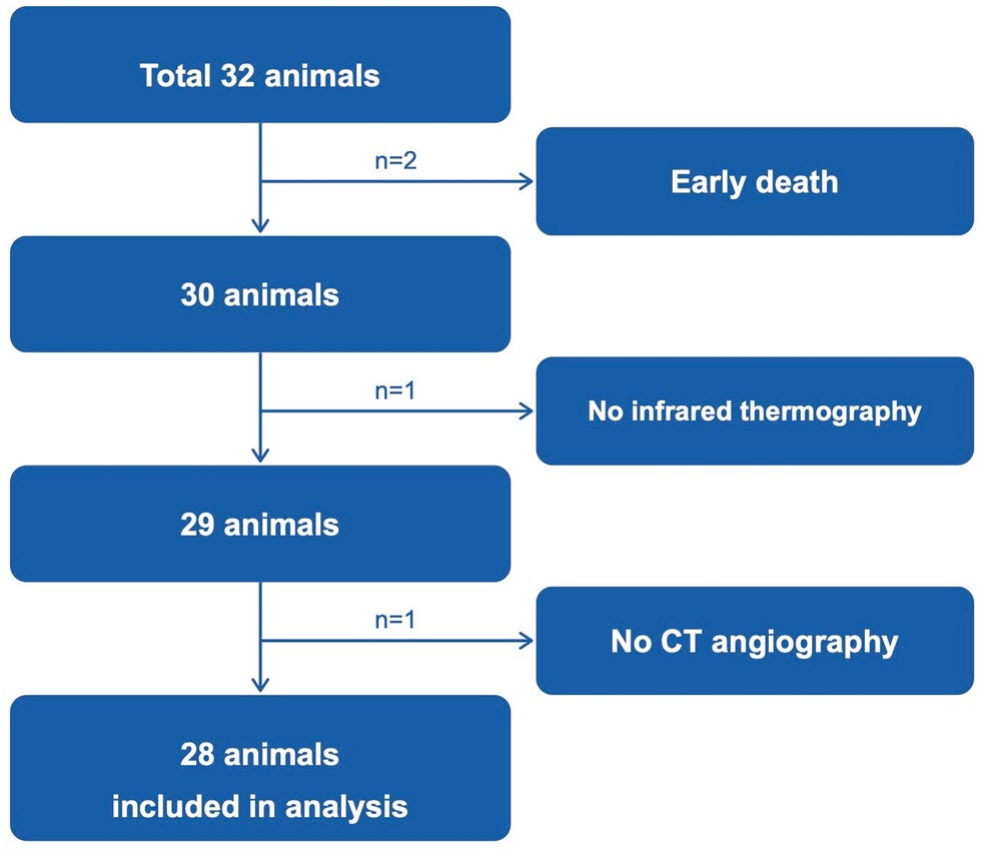
Study flowchart and inclusion of animals. Flowchart illustrating animal inclusion and exclusion. A total of 32 pigs were investigated in the experimental polytrauma model. Two animals were excluded due to early death during hemorrhagic shock, one due to missing infrared thermography data, and one due to missing CT angiography, resulting in 28 animals included in the final analysis

In the current study two animals were excluded due to early death during hemorrhagic shock. One animal each was excluded due to missing CT-imaging or FLIR data. A flowchart of the excluded animals is displayed in Fig. 1.

### CT angiography and spectral imaging

Imaging was performed during the resuscitation phase using a detector based spectral CT scanner (Spectral CT 7500, Philips Healthcare, Amsterdam, The Netherlands). Four whole-body CT scans without dose reduction were performed: native, arterial phase (automatic trigger on descending aorta), venous phase (4 min post contrast) and late phase (10 min post contrast). A total of 92 ml of iodine contrast agent (Ultrasvist 300, 50 ml 100% + 70 ml 60/40% mix with saline) were injected using a power injector at a rate of 5 ml/s, followed by saline flush of 40 ml. For each animal, femoral arterial vessel diameters were measured bilaterally in identical anatomical segments using axial CT reconstructions. Vascular compromise was defined as a vascular lesion caused by puncture, perivascular hematoma, or pseudoaneurysm, resulting in a ≥ 50% reduction in vessel diameter compared with the contralateral side. Iodine concentration was quantified using the spectral iodine density maps reconstructed on the CT scanner. Image analysis was performed using DeepUnity Review (Dedalus, Bonn, Germany). Regions of interest were placed distal to the catheterized segments at similar positions to diameter measurements.

### Infrared thermography

**Fig. 2:**
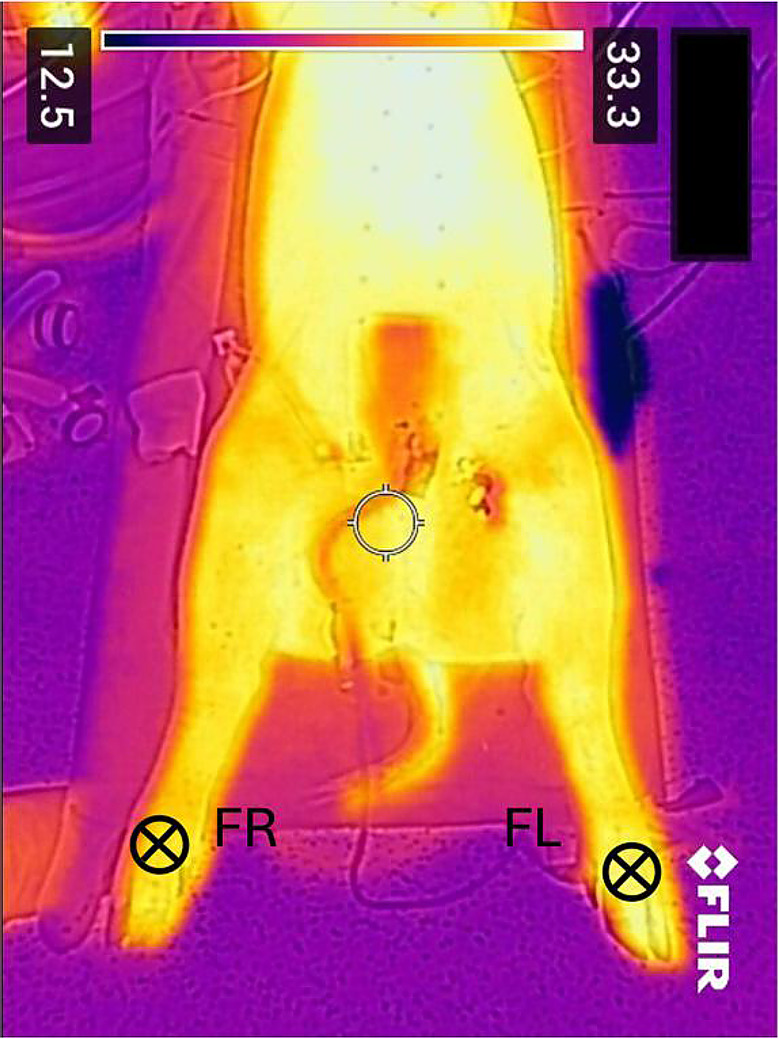
Infrared thermography of hind limbs and definition of regions of interestRepresentative infrared thermographic image of a porcine hind limb acquisition. Both hind feet were imaged simultaneously under standardized environmental conditions. Regions of interest (ROIs) were placed on the surface of the right foot (FR) and left foot (FL). The inter-limb temperature difference was defined as the absolute difference between mean temperatures of both ROIs

Infrared thermography (IRT) was performed at the beginning of the resuscitation phase prior to blood reinfusion using a handheld FLIR C5 thermal imaging camera (FLIR Systems, Wilsonville, OR, USA). The device has a thermal sensitivity (NETD) < 70mK, an infrared resolution of 160 × 120 pixels, and a visual image resolution of 5 megapixels. It operates in a spectral range of 7.5–14 μm, with a field of view of 54° × 42° and a fixed-focus lens. The total weight of 190 g enables easy handheld operation and rapid bedside use. Thermal images were acquired at a fixed distance of 1 m under constant ambient temperature (21 ± 1 °C) and relative humidity (50 ± 5%). To minimize reflections or artifacts, imaging was performed in a controlled laboratory environment without direct heat sources or airflow. Both hindlimbs and feet were captured simultaneously in each frame. The thermographic images were analyzed using FLIR Thermal Studio software (Teledyne FLIR LLC, US). As regions of interest, both limbs were measured. Position of measurement is displayed in Fig. 2. The inter-limb temperature difference was defined as the absolute difference between right (foot right (FR)) and left (foot left (FL)) mean temperatures.

### Statistical analysis

Statistical analysis was performed using R-Studio (R Foundation for Statistical Computing, Vienna, Austria, Version 2024.12.1 + 563). Continuous variables are reported with mean and standard deviation (SD), or 95%-Confidence interval in graphical presentation, categorical variables as count and percentage. Group comparisons between vascular lesion vs. non-lesion pigs were performed using the Welch t-test. Correlation analyses between vessel diameter reduction, iodine uptake, and temperature difference were performed using the Pearson correlation coefficient. Correlation strength was classified according to Cohen-classification [23]. In addition, simple linear regression was used to quantify the relationship between stenosis degree (independent variable) and temperature difference (dependent variable), and between iodine uptake and diameter reduction. To evaluate the predictive value of thermal asymmetry for vascular lesions, receiver operating characteristic (ROC) analysis was performed, and the area under the curve (AUC), 95% confidence interval, sensitivity, specificity, and optimal cutoff were calculated. Statistical significance was set at *p* < 0.05 for all analyses.

## Results

In the current study, 28 pigs were analyzed. Six out of 28 pigs (21.5%) fulfilled the criteria for a relevant vascular compromise. In this group, the mean reduction of vessel diameter was 68% (± 18%).

**Fig. 3:**
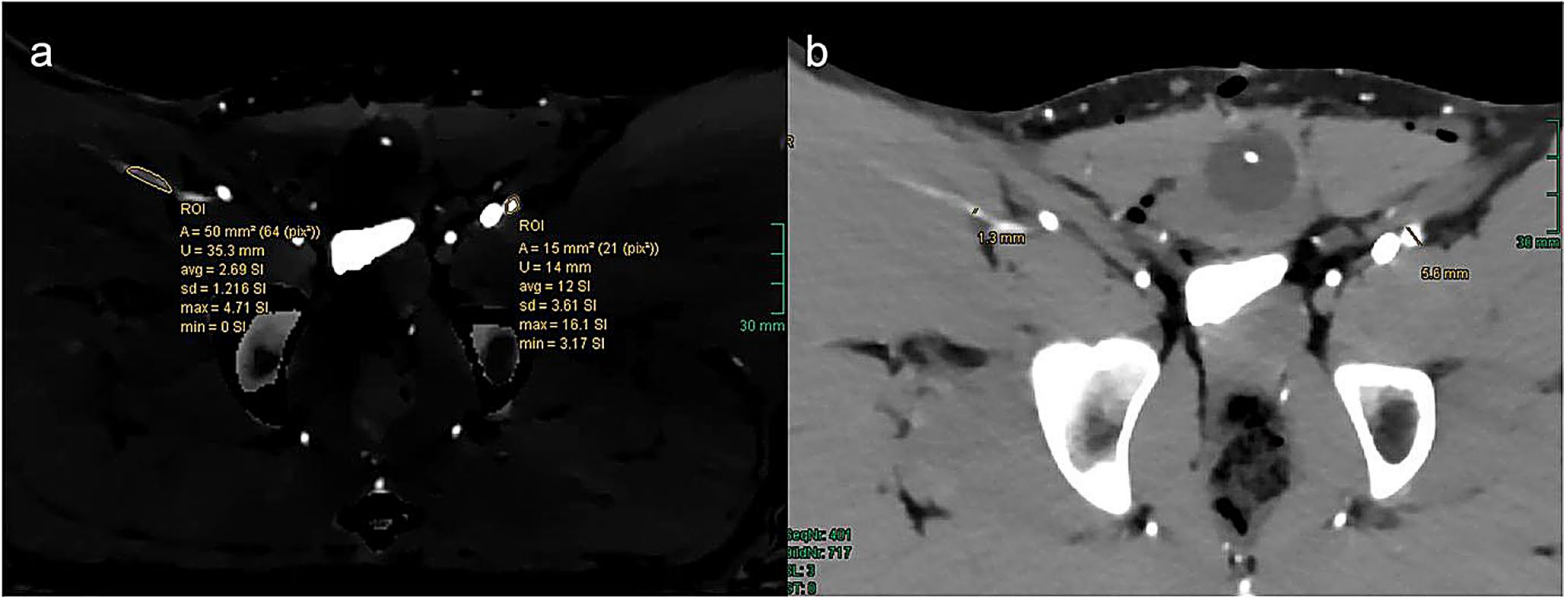
Multimodal imaging of femoral vascular compromise. (**a**) Spectral detector CT iodine density map showing reduced iodine uptake distal to a right-sided femoral arterial stenosis. Regions of interest were placed bilaterally to quantify tissue iodine concentration. (**b**) Corresponding axial CT angiography image demonstrating a relevant diameter reduction of the right femoral artery compared to the contralateral side

Mean vessel diameter on the right side was 4.64 (± 1.63) mm in the non-lesion and 1.75 (± 1.13) mm in the vascular lesion group, respectively (*p* < 0.001). A representative visualization of the reduced vessel diameter and iodine uptake is provided in Fig. 3.

Four out of six pigs with vascular compromise in CT were from a group with hemorrhagic shock. The remaining two cases occurred in animals assigned to the tissue trauma group, without hemorrhagic shock. Mean MAP in this group was 32.3 mmHg. In the non-lesion group 9 pigs were from a group with hemorrhagic shock and 11 pigs from a group without hemorrhagic shock. Mean MAP in the non-lesion group was 55.9 mmHg.

### Temperatures of extremities

**Fig. 4:**
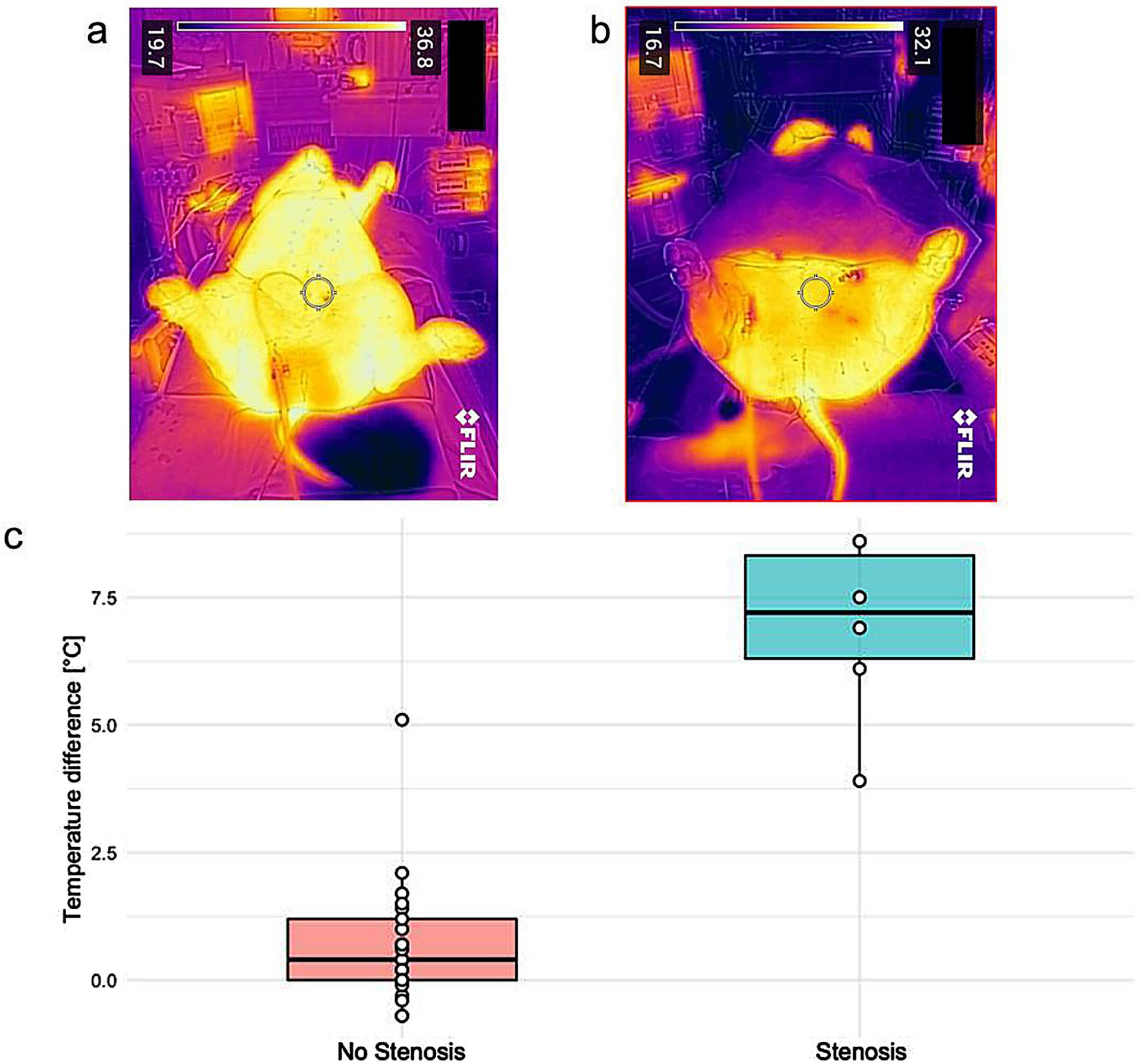
Thermographic findings and temperature asymmetry in animals with and without vascular compromise. (**a**) Representative infrared thermographic image of an animal without femoral vascular stenosis showing symmetrical temperature distribution of both hind limbs. (**b**) Representative infrared thermographic image of an animal with CT-confirmed right-sided femoral arterial stenosis demonstrating marked hypothermia of the affected limb compared to the contralateral side. (**c**) Boxplot showing inter-limb temperature differences between animals without femoral vascular stenosis and those with CT-confirmed stenosis ≥50%. Animals with stenosis demonstrated significantly higher temperature asymmetry compared to non-stenotic animals (p<0.001). Individual data points are displayed

In the vascular lesion group, a mean temperature difference between left and right of 6.93 (± 1.78) °C was evident. Mean temperature of the feet was 30.30 °C on the left and 23.37 °C on the right side. In the non-lesion group only a slight temperature difference with 0.71 (± 1.27) °C was evident. This results in a significant difference between both groups (*p* < 0.001) (Fig. 4). Interestingly, one pig had a temperature difference of 5 °C, but no significant diameter reduction on the right side (3.3 mm vs. 4.8 mm) was evident. However, SDCT analysis revealed a markedly reduced iodine uptake on the affected side (3.60 mg/ml vs. 7.52 mg/ml).

### Correlation temperature difference and diameter reduction

**Fig. 5:**
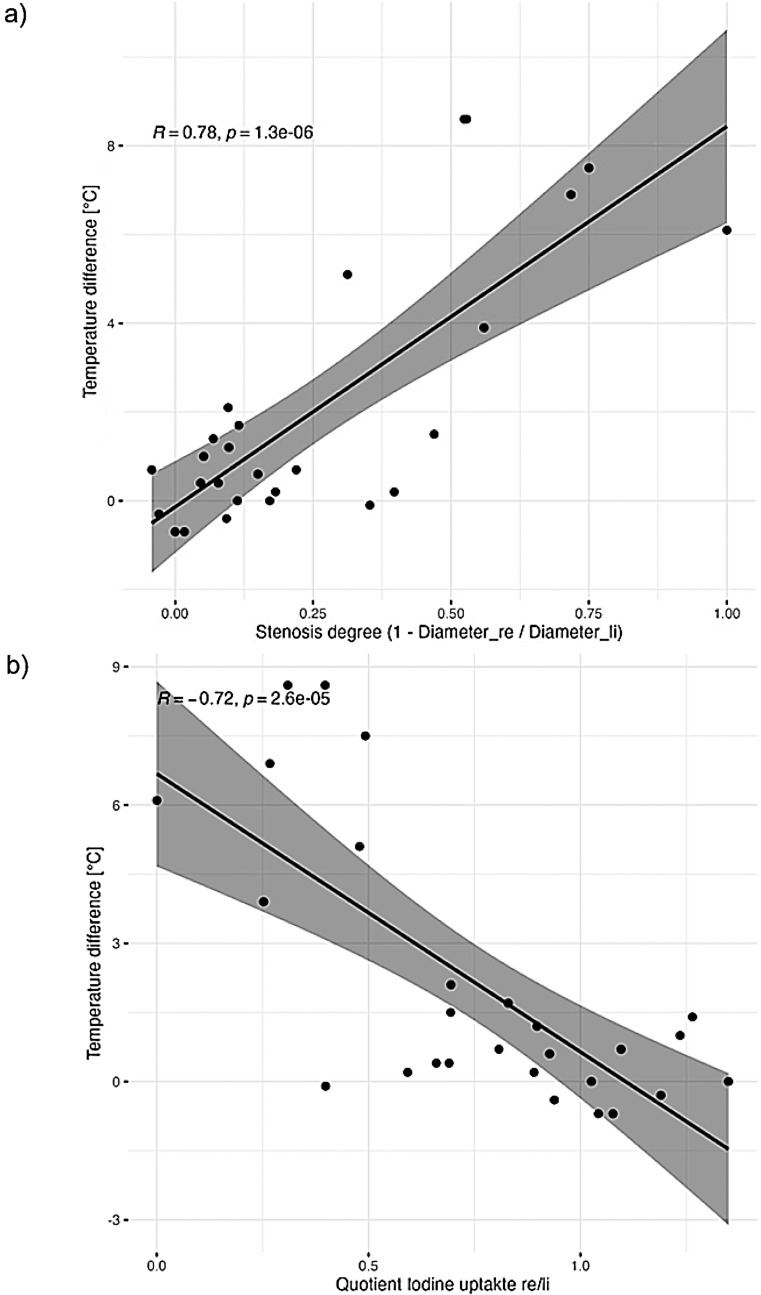
Correlation between vascular stenosis, iodine uptake, and temperature difference. (**a**) Scatter plot illustrating the positive correlation between femoral artery diameter reduction and inter-limb temperature difference (r = 0.78, p < 0.001). (**b**) Scatter plot showing the inverse correlation between SDCT-derived iodine uptake ratio (right/left) and temperature difference (r = –0.72, p < 0.001). Solid lines represent linear regression with 95% confidence intervals (shaded areas)

A strong positive correlation was found between diameter-based vascular stenosis and foot temperature difference (*r* = 0.785, *p* < 0.001). Linear regression analysis revealed a significant effect of stenosis on temperature difference (β = 8.57 °C, *p* < 0.001), with a model R² of 0.62. This indicates that each 10% increase in stenosis degree was associated with an average increase of approximately 0.86 °C in foot temperature difference (Fig. 5a).

### Correlation temperature difference and iodine uptake

In the vascular lesion group, mean iodine uptake behind the catheter was 3.21 (± 2.05) mg/ml on the right and 11.15 mg/ml on the left side. In a comparable manner, as diameter reduction, iodine uptake showed a strong negative correlation with temperature difference (*r*=-0.717, *p* < 0.001) (Fig. 5). 

### Correlation between diameter reduction and iodine uptake

To determine, whether iodine uptake is a reliable parameter for flow reduction, correlation analysis between diameter reduction and iodine uptake was performed. A very strong correlation between both parameters was observed (*r* = 0.801, *p* < 0.001). Linear regression model revealed a R^2^ of 0.64.

### ROC analysis of temperature difference as diagnostic tool

**Fig. 6:**
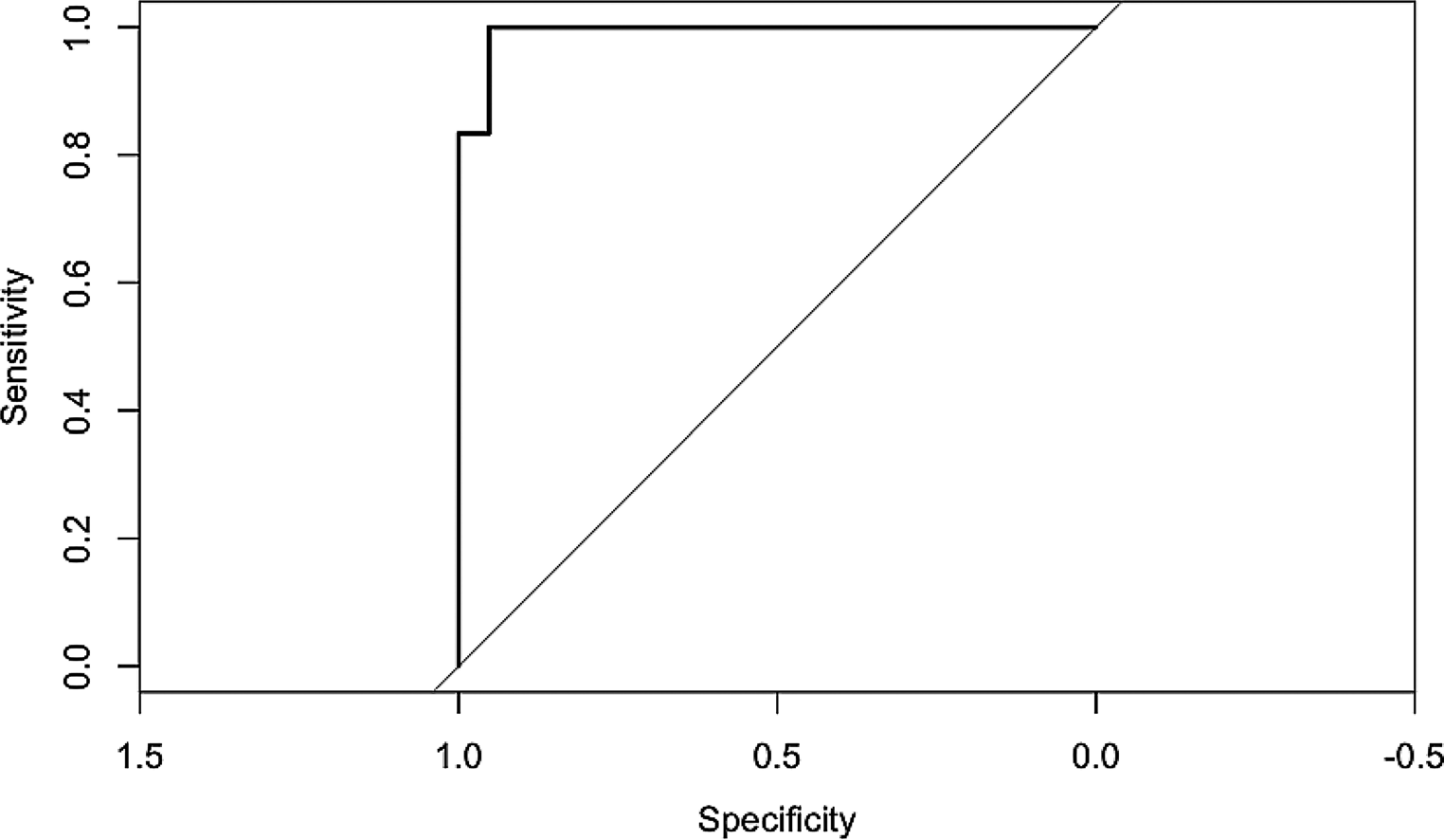
Diagnostic performance of temperature difference for detecting femoral vascular stenosis. Receiver operating characteristic (ROC) curve evaluating inter-limb temperature difference for detection of femoral arterial stenosis ≥50%. The area under the curve (AUC) was 0.99 (95% CI 0.97–1.00, p < 0.001). A threshold of 3 °C achieved 100% sensitivity and 95.2% specificity

Receiver operating characteristic (ROC) analysis demonstrated excellent diagnostic performance of the temperature difference for detecting vascular lesion. The area under the curve (AUC) was 0.9921 [95% CI 0.9701 to 1.0, *p* < 0.001] (Fig. 6). At an optimal threshold of 3 °C temperature difference, the model achieved a sensitivity of 100% and a specificity of 95.2%.

This indicates that, in this dataset, a temperature difference ≥ 3 °C discriminates between stenotic and non-stenotic animals with high accuracy. For the cutoff of 3°C the positive predictive value (PPV) was 85.7% and the negative predictive value (NPV) was 100%.

## Discussion

Vascular occlusion and undetected vascular injuries are major causes of morbidity in trauma and emergency surgery worldwide. Peripheral arterial injuries are associated with high rates of secondary complications, including compartment syndrome, tissue necrosis and infection. In literature, amputation rates between 9 and 50% have been reported after traumatic vascular lesions [24–28]. Delayed diagnosis and treatment may result in higher amputation rates up to 86% [27]. Furthermore, delayed recognition may increase the likelihood of prolonged intensive care unit (ICU) stay, repeated operations, and long-term functional impairment. Beyond the individual burden for patients, these complications generate considerable healthcare costs due to extended operative time, resource-intensive postoperative monitoring, and lengthy rehabilitation [29].

Given the relevance of early detection of vascular compromise, our main findings in the current study are as follows:

1. Acute femoral vascular compromise occurred in a relevant proportion of animals and was associated with marked perfusion impairment.
2. Infrared thermography showed a strong diagnostic signal for the detection of limb ischemia.
3. Inter-limb temperature differences strongly correlated with both the degree of CTA-derived arterial diameter reduction and distal tissue perfusion.
4. Dual-layer spectral CT–derived iodine uptake showed a strong correlation with anatomical stenosis severity and served as a robust quantitative surrogate marker of reduced tissue perfusion.

Determination of vascular compromise using CT angiography (CTA) is an established tool in radiology. Recent studies showed that stenosis detection with CT is safe and reliable compared to Digital subtraction angiography (DSA) [10, 11, 17, 30]. For CTA usage in vascular stenosis a sensitivity of 85–90% has been revealed [31, 32]. In literature, a diameter reduction of ≥ 50% is considered as hemodynamically relevant and therefore suspected as cutoff [30].

The mean temperature difference of 6.93 °C (± 1.78 °C) observed in the vascular lesion group compared to 0.71 °C (± 1.27 °C) in the non-lesion group represents a clinically significant difference. This aligns with physiological principles that reduced arterial perfusion leads to decreased tissue metabolism, resulting in measurably lower surface temperatures [21]. Previous studies have established the utility of thermography in chronic peripheral artery disease, with temperature differences of 1–3 °C considered clinically significant [33–35]. In the current study an ideal threshold of 3 °C was identified. The larger temperature differences observed in this study may reflect the acute nature of the vascular occlusion and correlate with the severity of stenosis. Furthermore, the concurrent hemorrhagic shock state may pronounce the temperature difference. Importantly, IRT was obtained before blood retransfusion, indicating that the observed thermal asymmetries reflect the physiological state during the shock rather than differential limb rewarming during blood reinfusion. Most studies in the literature were performed in chronic situation. Collateral circulation development and metabolic adaptation may partially compensate for reduced flow, resulting in smaller temperature gradients. However, animal studies investigating complete occlusion using tourniquets or REBOA systems revealed similar temperature gradients measured by FLIR imaging [36, 37]. The 3 °C threshold identified in our study should therefore be considered a hypothesis-generating value rather than a validated clinical decision threshold.

One notable finding was an animal with a 5 °C inter-limb temperature difference despite only minimal diameter reduction on CT. In this case, SDCT-derived iodine uptake was substantially reduced on the affected side, suggesting that the observed thermal asymmetry may reflect functional tissue hypoperfusion rather than macroscopic vessel narrowing alone. This finding may represent a perfusion–anatomical dissociation. While this observation raises the possibility that IRT may capture physiologically relevant perfusion abnormalities beyond gross anatomical stenosis, alternative explanations such as measurement variability or local external warming effects cannot be excluded.

The correlation coefficient observed in this study (*r* = 0.785 for degree of stenosis vs. temperature) is comparable to those reported for other non-invasive vascular assessment tools, suggesting that thermography performs favorably in comparison to established modalities in this specific clinical context [35].

Perfusion measurement using spectral CT is a quite novel tool and rarely described in literature. However, some studies suggest this as a promising diagnostic tool/device. The strong correlation between iodine uptake and diameter reduction (*r* = 0.801, *p* < 0.001) validates spectral CT as a quantitative tool for assessing flow compromise. The marked difference in iodine uptake distal to the compromise (3.21 ± 2.05 mg/ml) compared to the contralateral side (11.15 mg/ml) reflects the reduced contrast delivery to tissues with compromised arterial inflow. This represents a significant advantage over conventional CT angiography, which provides primarily anatomical information about vessel patency without quantifying the functional impact on tissue perfusion. The correlation between iodine uptake and temperature difference (*r* = -0.717, *p* < 0.001) supports the physiological link between reduced perfusion and decreased tissue temperature. This finding suggests that both modalities are measuring impaired tissue perfusion.

One advantage of IRT is, that it is a handheld tool, which can be easily and fast used in clinical practice. Since it is radiation free and usable without patient contact, it can be performed by bystanding persons. As CTA is regularly available in major trauma centers, it might be a possibility that patients are assessed prior to CT scanning with IRT to decide, if angiography of extremities is necessary. IRT may therefore be used as a quantitative extension of the classical clinical sign of poikilothermia rather than a replacement of established diagnostic pathways. Another possibility might be the use in battlefield. Since vascular injuries are frequent combat injuries, this might be an easily accessible tool in the decision process whether a CTA is needed [38].

The current study has several limitations. One of them being that IRT surface temperatures can be influenced in different manners, especially in the polytrauma setting. In polytrauma patients it is recommended to perform immediate indirect warming, e.g. with warm blankets or indirect forced warming [39, 40]. This might give false results. Furthermore, internal factors such as infection and inflammation may also influence results [20].

The occurrence of vascular lesion was not planned in the current study and therefore the reason remains unclear. No randomization could be performed. Both may represent a relevant methodological issue. With only 6 animals in the vascular lesion group, this may be underpowered. Larger studies are needed to confirm these findings. Furthermore, we assumed vascular lesion by CTA. Since DSA is still the gold-standard in literature, there might be a small bias [41–43].

## Conclusion

In conclusion, the current study suggests that thermographic imaging may serve as an adjunctive tool for the detection of vascular compromise in a porcine model of hemorrhagic shock. Additionally, spectral CT based quantification of iodine uptake showed a strong association with both anatomical stenosis and temperature differences, supporting its role as a quantitative perfusion-related parameter. Together, these findings suggest that both techniques may have potential as adjunctive tools in assessing vascular damages or injuries in trauma patients. Further validation in larger and clinically oriented studies is needed.

## Data Availability

The datasets used and/or analyzed during the current study are available from the corresponding author on reasonable request.
